# Correction: A computational modeling approach for predicting multicell spheroid patterns based on signaling-induced differential adhesion

**DOI:** 10.1371/journal.pcbi.1011166

**Published:** 2023-05-22

**Authors:** Nikita Sivakumar, Helen V. Warner, Shayn M. Peirce, Matthew J. Lazzara

The following information is missing from the Funding statement: This work was supported by funding from the National Institute of General Medical Sciences Grant No. GM140008 (SPC).

In the “Cell lines and propagation” subsection of “Materials and methods”, there is an error where fibroblast cells were incorrectly referred to as adipocytes. The correct sentence is: L929 murine fibroblasts engineered with a bidirectional cell -cell signaling circuit based on the synthetic notch (synNotch) receptor system [2] were generously provided by Dr. Wendell Lim (University of California, San Francisco).
