# Supplementary figures and images for: A computational modeling approach for predicting multicell spheroid patterns based on signaling-induced differential adhesion
Source: PLoS Comput Biol. 2022 Nov 28;18(11):e1010701. doi: 10.1371/journal.pcbi.1010701 (PMC9747056; doi:10.1371/journal.pcbi.1010701)

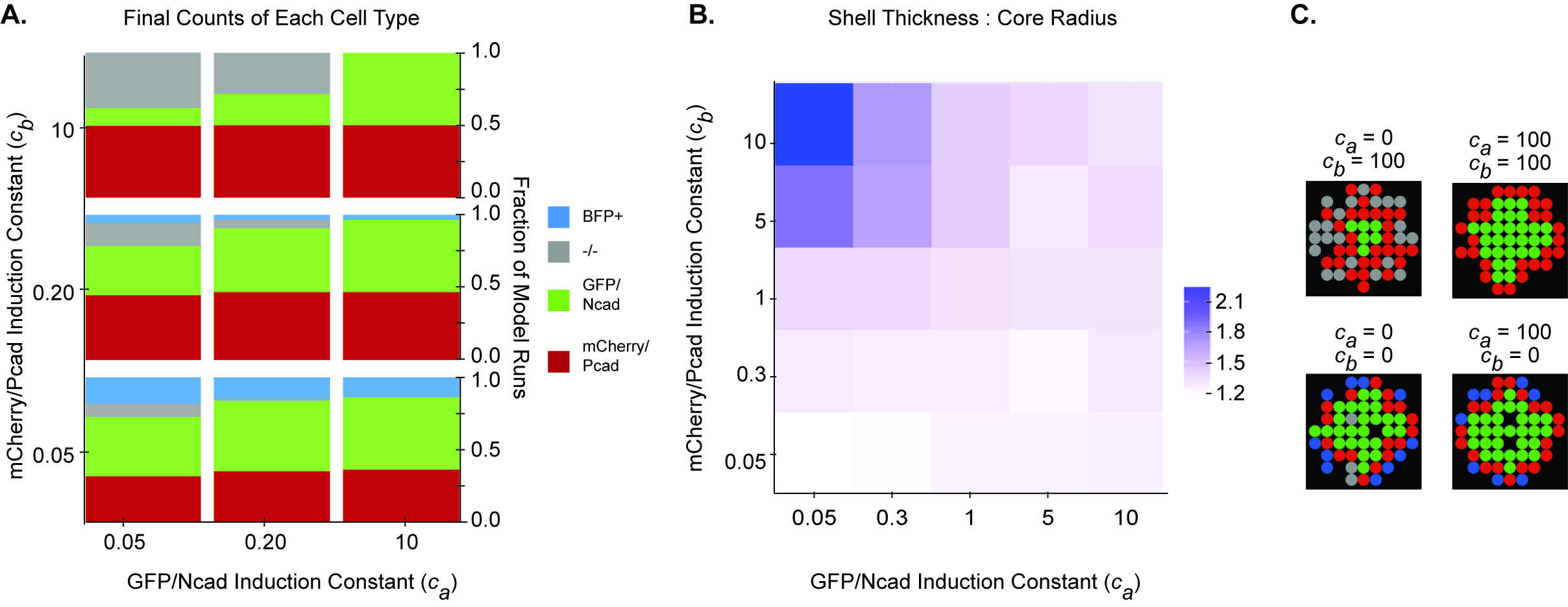

Supplement: S1 Fig — (A) The average fractions of BFP+, -/-, GFP/Ncad, and mCherry/Pcad cells in spheroids were calculated for each parameter combination. (B) The cadherin induction constants (ca, cb) were simultaneously varied across values of 0.05, 0.3, 1, 5, and 10 for Ruleset 3B (bidirectional signaling core/shell ruleset) in the 2D ABM. For each parameter combination, simulations were seeded with 200 cells at a 1:1 BFP+:-/- ratio and run 100 times, each for 100 timesteps. (C) Representative images of spheroids from extremes of 9E are shown. (TIF) [file pcbi.1010701.s001.tif]

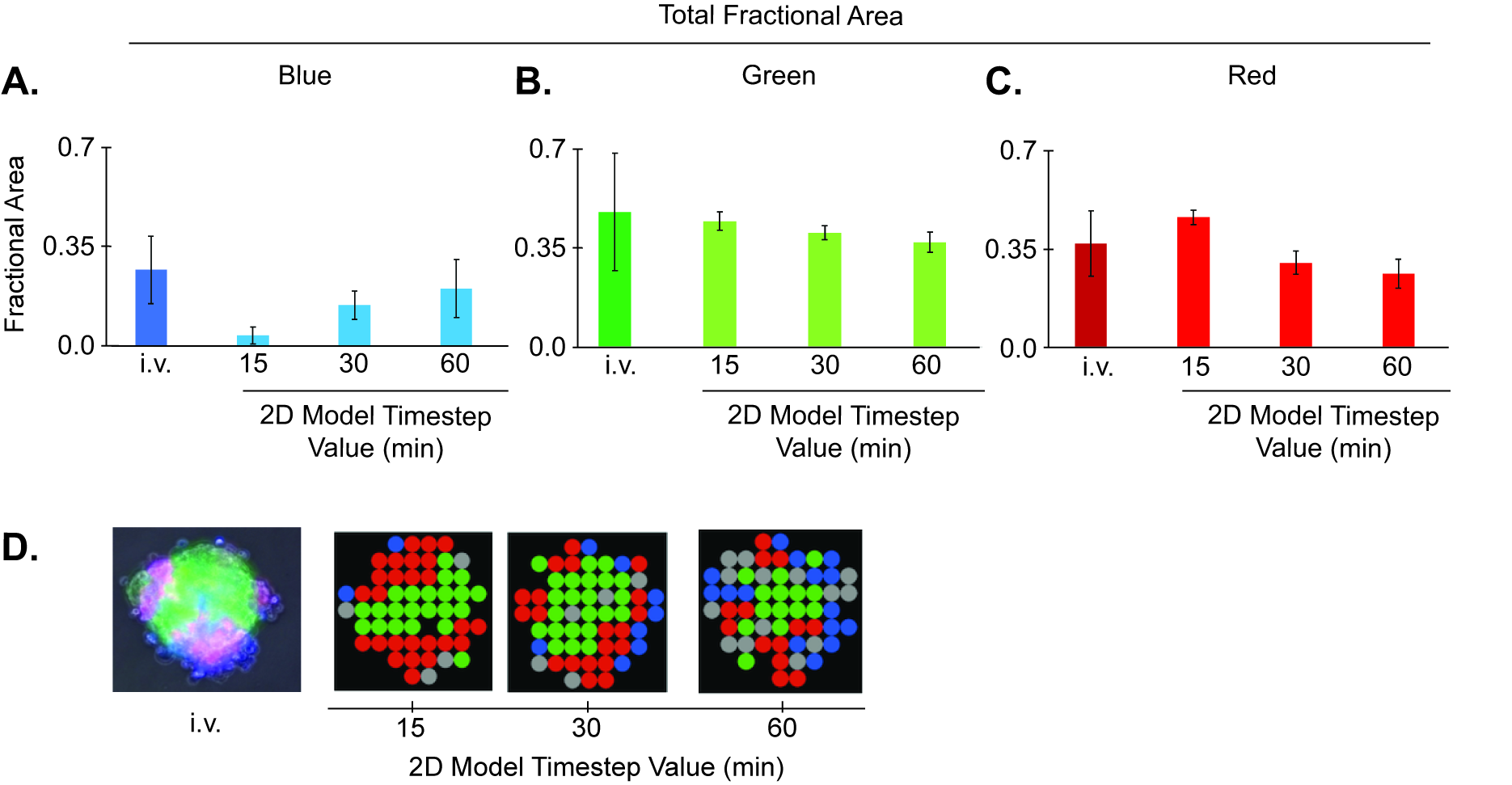

Supplement: S2 Fig — Comparisons were made of (A) blue, (B) green, and (C) red fractional areas between in vitro (i.v.) experiments and 2D ABM predictions, where the ABM was run for three different timestep values to represent experiments seeded with 200 cells at 1:1 ratio of BFP+ and -/- cells. ABM-predicted fractional areas for the 2D model conditions were calculated as an average of 100 model runs, each run for 200, 100, and 50 total timesteps for the 15-, 30-, and 60-min time steps, respectively. Fractional areas from in vitro images were calculated from a sample of 10 images. Error bars indicate standard deviation. (D) Representative images of ABM predictions for each timestep are compared against a representative experimental image. (TIF) [file pcbi.1010701.s002.tif]
